# Supplementary material for: Visuo-motor integration, vision perception and attention in mTBI patients. Preliminary findings
Source: PLoS One. 2021 Apr 27;16(4):e0250598. doi: 10.1371/journal.pone.0250598 (PMC8078787; doi:10.1371/journal.pone.0250598)
Supplement: S1 Appendix — (PDF) [file pone.0250598.s001.pdf]

S1 Appendix. Convergence Insufficiency Symptom Survey (CISS).

Convergence Insufficiency Symptom Survey (CISS)

1. Do your eyes feel tired when reading or doing close work
2. Do your eyes feel uncomfortable when reading or doing close work
3. Do you have a headache when reading or doing close work
4. Do you feel sleepy when reading or doing close work
5. Do you lose concentration when reading or doing close work
6. Do you have trouble remembering what you have read
7. Do you have double vision when reading or doing close work
8. Do you see the words move, jump, swim, or appear to float on the page when reading or doing close work
9. Do you feel like you read slowly
10. Do your eyes ever hurt when reading or doing close work
11. Do your eyes ever feel sore when reading or doing close work
12. Do you fell a "pulling" feeling around your eyes when reading or doing close work
13. Do you notice the words blurring or coming in and out of focus when reading or close work
14. Do you lose your place while reading or doing close work
15. Do you have to re-read the same lime of words when reading

Likert scale:

1 - Never 2 - Rarely 3 - Sometimes 4 - Often 5 - Always
