# Supplementary material for: Visuo-motor integration, vision perception and attention in mTBI patients. Preliminary findings
Source: PLoS One. 2021 Apr 27;16(4):e0250598. doi: 10.1371/journal.pone.0250598 (PMC8078787; doi:10.1371/journal.pone.0250598)
Supplement: Attachment — Submitted filename: Response to Reviewers.docx [file pone.0250598.s003.docx]

# Response to Reviewers

# Reviewer #1:

*This empirical study examined the visuomotor skill, visual motion perception, and visuospatial attention in young adults both with and without a history of minor traumatic brain injury (mTBI). They observed significant impairments in the visuomotor integration as well as visual motion perception in the mTBI group relative to the control group. The authors discuss their findings in the context of previous findings around brain injury affecting visual and visuomotor areas of the brain. I found literature review and rationale solid, the methods sound, the discussion thorough, and the writing clear. I have a few concerns around clarity of the hypotheses (not stated) and data analysis.*

**We thank the reviewer for his/her suggestions, we respond to each comment below.**

*Specific comments:
Intro: Line 45: This would be an appropriate place to spell out your hypothesis, given that this appears to be set up as a hypothesis-driven study and there are adequate previous findings to set up a novel set of hypotheses for the present study. There are two references to hypotheses in the text (data analysis, line 205), but nothing actually spelled out****.*** *Indeed, the final concluding paragraph is the first place one sees a sentence describing a hypothesis!*

**We specify better in the Introduction the aim and hypothesis of our study (see p. 5 lines 100-113).**

*It is not until you get to the methods section that you realize the median time since concussion is many weeks. Thus, this is a study that focusses on the ‘persistent symptom’ situation (which some authors refer to as post-concussion syndrome, but there is a lack of clarity in the field at the moment around the parameters for these categories). Perhaps the authors should have a few sentences in the introduction on this, and make it clear in the abstract that this is a study using a persistent symptom population versus an acute post-injury population. This would be useful to the reader.*

**We thank the reviewer for the comment, we have added in the Introduction and in the abstract a comment on the specific population that we refer to (see p.5 lines 105-110).**

Methods/results:
Were there any initial analyses of sex-related differences in your data set. Or, given the sample sizes, was there not enough power for such analyses? Regardless, some mention should be made if an analysis was done or not. This is now standard procedure in biomedical research.

**We confirm that it was not possible to evaluate gender differences because of the small sample size included in the study (only 2 females in the control group). We included a paragraph mentioning this limitation in the method section (p. 5 line 118).**

*Were there any tests applied to examine dependent variable values as a function of time since concussion, within the concussion group? Given the range (if I’m interpreting the 148 days IQR 46-256 correctly) in time since concussion as well as the range in symptomology, I and other readers would be interested if there is a time effect within the concussion group for your measures.*

**We thank the reviewer for the comment. We included an analysis based on Spearman’s rho correlation in the results section and a table (Table 1) showing the correlation between the measures used in the present study and time since concussion and symptoms severity. Although the limitation due to the small sample size, the results showed that time since the concussion is related and symptoms severity are correlated with visuomotor and motor ability assessed with VMI-6 (p. 13 line 312 and p.14).**

*Discussion
On line 266 there is mention of correlations. This is the first and only reference to correlations between measures. Should a description of this analysis and findings be in the text somewhere?*

**We thank the reviewer for the comment. We included in the results section the correlations based on Spearman’s rho correlation analysis, and the values are visible in Table 1 (p. 13 line 312 and p.14).**

*Lines 282-284 seem oddly placed and redundant, since there is a conclusions section at the very bottom. Also, the phrase eye-motor coordination is confusion, since one is not sure if you’re referring to eye-hand coordination or oculomotor control. But this whole paragraph could be deleted.*

**We thank the reviewer for the comment. We agree that the paragraph is redundant, therefore we have deleted it, as suggested.**

*Minor:
1.Abstract: “These preliminary findings suggest that the temporary brain insults deriving from mTBI compromise seriously fine motor and visuo-motor integration together with form recognition and visuo-spatial attention.”
Phrasing odd (it sounds like you’re referring to ‘seriously fine motor’ and then there’s no noun). Perhaps just ‘…**mTBI compromise fine motor skills, visuomotor integration, form recognition, and visuo-spatial attention’.*

**We thank the reviewer for the suggestion. We corrected the abstract accordingly.**

*2. Abstract:” The unexpected results concerning motion perception could be explained by the position of the stimulus that interested the fovea parafovea whilst in previous studies the stimuli were presented in the peripheral visual field.” Something is missing in this sentence.*

**We thank the reviewer for the comment, we deleted the sentence and changed the last paragraph indicating the new results obtained from correlation analysis.**

*3. Intro, line12: “…such as easily fatigued…”, such as being easily fatigued*

**We thank the reviewer for the comment we have corrected the sentence (p.3, line 61).**

*4. Discussion, line 253: “..that required a good space-control..”. Unclear what this means.*

**We clarify the sentence (p. 15, line 356) as follows: “On the contrary, they did not show any difficulties when drawing 3D figures or figures with intersections that required good visuo-spatial ability but which were less demanding in simultaneous control of information.”**

*5. Discussion line 280: “… confirmed that already teo weeks post-injuries…” ten weeks post-injury (presumably)*

**We thank the reviewer for the comment we have corrected the typo (p. 15, line 356).**

# Reviewer #2

*This study aims to better understanding the mechanisms that visuo-motor imparments in mild traumatic brain injuries (mTBI) patients. From a behavioural approach, visuo-motor integration of fine motor abilities, motion perception and visuo-spatial attention were measure with several tests. The results coming from 11 mTBI patients were compare to a healthy control group. Dealing with the visuo-motor integration, the form recognition, the motor deficits and the visuo-patial attention, the results are in line with the authors’ main hypotheses and highlight an impairment of these functions in mTBI patients. At the opposite, motion perception was not altered in mTBI patients.
Overall, this study is interesting. The scientific questions raised in their clinical and fundamental context appear relevant. Nevertheless, some major points could significantly improve this work.*

*1/ The introduction section has a very clinical coloring. Yet the study is conducted with only 11 patients. This is not crippling in itself, but a state-of-the-art approach more focused on the field of visuo-motor control and its underlying mechanisms seems to me more appropriate with the experimental content. Some references of works and models developed by M.Desmurget, Y.Rossetti, or C.Prablanc… appeared missing to me. Moreover, the results of present study could be discussed in the light of these references which are lacking, in order to make the discussion even more attractive.*

**1/We thank the reviewer for the suggestions. We included more information and references on the field of visuo-motor control in the introduction (p. 4 lines 83-90) and discussion (see p. 14-15, lines 335-362).**

*2/ - My major concerns relate to the presentation of the results, the style of chosen figures, the reported variability, and the nomenclatures displayed. Indeed, the graph bar are very bad representation for a sample of 11 or 10. They do not make it possible to account for variability and distribution. Whisker plot or scatter plot appear much more relevant.
- Graphical visualization of statistical effects would greatly assist the reader.
- Fig 1 and 2 should be grouped together in a same figure with two panels.*

**2/ We thank the reviewer for the suggestions. We modified the figures as requested.**

*3/ The discussion section appeared really descriptive to me. The parallel between the results and the theoretical models of visuomotor functions could be reinforced.*

***3/ We agree with the reviewer comments, we would be more cautious in interpreting the results because of the small sample size, however, we try to improve the discussion section and reinforce the theoretical models of visuomotor functions as suggested* (see p. 14-16)*.***

*4/ What are the authors' recommendations for perspective of such a work in light with these results?*

***4/We included final recommendations in the discussion session as follows:***

***“Although preliminary, the findings suggest the importance to further investigate how the visuomotor impairments in mTBI could be related to symptoms severity and postconcussion syndrome. Moreover, the results suggested to include a larger sample to investigate possible confounding factors (e.g. the type of symptoms, the time since concussion and the demographic charactheristics of the patients, such as gender and age). If confirmed by future studies on larger samples, the results of this study may give indications on assessment strategies, useful for prognosis and for planning rehabilitation interventions in patients with mTBI.”***

*5/ In conclusion, this work is interesting and can be significantly strengthened. Concerning a major but acceptable limit, namely the sample, the work would be reinforced with a more detailed analysis and representation of the results, and not large averages in graphbars which may mask some fine effects to investigate or at least to comment on. “Preliminary” or “pilot study” could be clearly stated in the title.*

**5/ We thank the reviewer, we modified the description of the results including whisker plots and distribution plots and we included in the title the statement “Preliminary findings”.**
